# Characterization of Ablation Thresholds for 3D-Cultured Patient-Derived Glioma Stem Cells in Response to High-Frequency Irreversible Electroporation

**DOI:** 10.34133/2019/8081315

**Published:** 2019-04-28

**Authors:** J. W. Ivey, E. M. Wasson, N. Alinezhadbalalami, A. Kanitkar, W. Debinski, Z. Sheng, R. V. Davalos, S. S. Verbridge

**Affiliations:** ^1^School of Biomedical Engineering and Sciences, Virginia Tech-Wake Forest University, Blacksburg, VA 24061, USA; ^2^Department of Mechanical Engineering, Virginia Tech, Blacksburg, VA 24061, USA; ^3^Brain Tumor Center of Excellence, Comprehensive Cancer Center, Wake Forest Baptist Medical Center, Winston-Salem, NC 27157, USA; ^4^Virginia Tech Carilion Research Institute, Roanoke, VA 24061, USA; ^5^Department of Internal Medicine, Virginia Tech Carilion School of Medicine, Roanoke, VA 24016, USA; ^6^Faculty of Health Science, Virginia Tech, Blacksburg, VA 24061, USA

## Abstract

High-frequency irreversible electroporation (H-FIRE) is a technique that uses pulsed electric fields that have been shown to ablate malignant cells. In order to evaluate the clinical potential of H-FIRE to treat glioblastoma (GBM), a primary brain tumor, we have studied the effects of high-frequency waveforms on therapy-resistant glioma stem-like cell (GSC) populations. We demonstrate that patient-derived GSCs are more susceptible to H-FIRE damage than primary normal astrocytes. This selectivity presents an opportunity for a degree of malignant cell targeting as bulk tumor cells and tumor stem cells are seen to exhibit similar lethal electric field thresholds, significantly lower than that of healthy astrocytes. However, neural stem cell (NSC) populations also exhibit a similar sensitivity to these pulses. This observation may suggest that different considerations be taken when applying these therapies in younger versus older patients, where the importance of preserving NSC populations may impose different restrictions on use. We also demonstrate variability in threshold among the three patient-derived GSC lines studied, suggesting the need for personalized cell-specific characterization in the development of potential clinical procedures. Future work may provide further useful insights regarding this patient-dependent variability observed that could inform targeted and personalized treatment.

## 1. Introduction

Glioblastoma (GBM), the most common and deadly primary brain tumor, has a dismal prognosis that has remained relatively unchanged despite decades of research [1]. A GBM tumor proves fatal within about 14 months even with multimodal intervention [2]. GBM tumors are treated with surgery followed by concurrent radiotherapy and adjuvant chemotherapy [3–5]. Neither single therapies nor treatments used in combination are curative and they are often debilitating to the patient. The failure of current treatments to greatly extend life expectancy is attributable, among other reasons, to several classes of therapy-resistant cells that propel tumor recurrence, which is nearly universal with GBM [6]. There exists a real need for next-generation GBM therapies, for use alone or in combination with current therapies, which can target the resistant cell populations and prevent tumor recurrence.

The highly therapy-resistant nature of GBM is due in large part to inter- and intratumor heterogeneity [7–12], which becomes a survival advantage for the tumor in resisting treatment [13, 14]. In addition, presence of blood brain barrier contributes to failure of most chemotherapies by preventing most therapeutic regents from penetrating into the tumor. Central to the highly heterogeneous makeup of a GBM tumor is its initiator cells that are the progenitors from which the many subclasses of cells that make up a tumor are derived. It has been hypothesized that just as an organ develops from stem cells, tumors such as GBM are similarly derived from a set of stem-like cells that make up a small percentage of the tumor but drive its development and progression [15]. There is still some controversy over whether these cancer stem cells are originator cells, responsible for the initiation and progression of the tumor or whether they are a product of tumor initiation and evolution [16]. However, regardless of their standing in the hierarchy of the tumor, they possess two characteristics that make them very important in the study of cancer therapies—their ability to self-renew and initiate new tumors and their ability to resist current cancer therapies. What have come to be known as glioma stem-like cells (GSCs) or brain tumor initiating cells (BTICs) are a class of cells in the brain that express high levels of stem cell markers involved in self-renewal as well as genes involved in neural stem cell (NSC) proliferation and differentiation.

In addition to their self-renewal properties, cancer stem cells have another important characteristic central to their role in the tumor hierarchy, that is, their high degree of resistance and hyperactive repair mechanisms. GSCs have been shown to have a variety of resistance mechanisms such as high expression levels of a variety of drug resistance genes (BCRP, MDR1). GSCs additionally show enhanced DNA repair capacities, linked to increased MGMT activity, increased expression of damage checkpoints, and highly activated apoptosis inhibitors [17–21]. Multiple molecular mechanisms have been identified in GSCs to mediate therapeutic resistance to cytotoxic therapies such as Notch [22], NF-*κ*B [23], EZH2 [24], and PARP [25]. Additional mechanisms of resistance may evolve from exposure to microenvironmental factors such as hypoxia [26, 27] and metabolic stress [28, 29]. These cells are able to maintain a tumor despite multimodal assaults by chemotherapies and radiation therapies [30, 31]. In general, cells are characterized as GSCs based on five criteria—ability for self-renewal, differentiation potential, high tumorigenicity, drug resistance, and radio-resistance [32, 33]. Together, these features of GSCs make them highly likely to significantly contribute to GBM recurrence and therefore a very attractive therapeutic target [34–36].

Because many methods of cancer treatment are ineffective for GSCs and because they are important in tumor progression and recurrence, a need exists for a different class of therapies that can work effectively against GSCs. Conventional therapy regimes often eliminate the bulk tumor while unintentionally concentrating the pool of GSCs that can propagate a tumor, resulting in near universal recurrence in GBM tumors [37]. We previously hypothesized that irreversible electroporation (IRE) is a therapy that holds promise for the targeting of GSCs. However, this hypothesis has not yet been directly tested for patient-derived GSCs that are highly relevant in human GBM progression and recurrence.

IRE is a nonthermal ablation mechanism used primarily for the treatment of surgically inoperable tumors [38–40]. In IRE treatment, two or more electrodes are inserted into the tumor and short (~100 *μ*s) high intensity monopolar pulses are applied to the tumor region [41]. These pulsed electric fields (PEFs) cause destabilization of the cell membrane which leads to cell death through loss of homeostasis [42–45]. A second generation of IRE uses shorter (~0.5-2 *μ*s) bipolar pulses delivered as a series of bursts. This treatment is known as high-frequency irreversible electroporation (H-FIRE). This therapy was developed due to its ability to reduce muscle contractions induced by IRE [46] and has been shown to elicit a more predictable response in heterogeneous tissue [47]. In addition, our previous studies have shown that by using H-FIRE; the efficacy of the treatment is enhanced in some cases for cells with a higher nuclear-to-cytoplasm ratio [48, 49]. It has also been shown that IRE and H-FIRE facilitate temporary disruption of blood brain barrier, allowing for penetration of therapeutic reagents [50, 51]. Disruption of blood brain barrier can be expected at the site of treatment, as well as in the sublethal surroundings, enabling the local delivery of drugs to the tumor site. Such combinatory treatments can provide a synergistic effect on eradicating the malignant cells.

Previous studies of the structural characterization of GSCs have reported atypical and enlarged nuclei in GSCs as well as irregular physical structure in other organelles [32, 52, 53]. Because we have previously demonstrated that nuclear size is strongly correlated with H-FIRE cell damage, we further hypothesize that not only will GSCs be susceptible to H-FIRE damage, but also this susceptibility may be enhanced compared with normal cell types such as astrocytes, which possess normal nuclear size. In this study, we therefore seek to determine the ability of H-FIRE therapy to ablate GSCs and the possibility of selective targeting of these cells. Such selectivity may be due to structural differences such as nuclear size or nuclear-to-cytoplasm ration (NCR), as well as yet-to-be discovered mechanistic differences in H-FIRE cellular responses.

## 2. Results

### 2.1. GSCs Exhibit Tumor-Characteristic Enlarged Nuclei Compared to Normal Astrocytes

**Figure 1:**
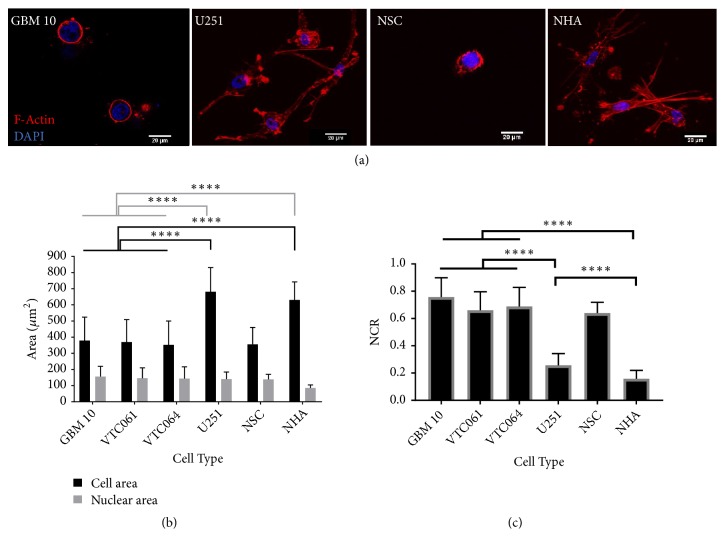
*Cell morphology characterization in collagen hydrogels.* (a) Confocal images of GSCs, U251, NSCs, and NHAs show differences in cell morphology of different cell types. (b) Cell areas for GSCs and NSCs are significantly smaller than U251 or NHAs (p < 0.0001). Nuclear areas for GSCs, U251 cells, and NSCs are enlarged compared to NHAs (p < 0.0001). (c) Calculation of NCR from confocal images shows a significantly higher NCR for GSC and NSC populations compared to both U251 and NHA (p < 0.0001). U251 have significantly higher NCRs than NHAs (^*∗∗∗∗*^p < 0.0001). (d) A comparison of circularity and aspect ratio across cell types illustrates the different cell morphology of GSCs and NSCs compared to U251 and NHA cells. GSCs and NSCs have a significantly higher circularity (^*∗∗∗∗*^p < 0.0001) than U251s and NHAs and a significantly lower aspect ratio (^*∗∗∗∗*^p < 0.0001). U251s and NHA have no significant difference in circularity or aspect ratio (p > 0.05).

The GSCs tested all grow as nonadherent tumorspheres, a common morphological characteristic of stem cells. To confirm that GSCs follow the trend of enlarged nuclear-to-cytoplasm ratio (NCR) that many other cancerous cells have been shown to exhibit, we used confocal imaging to determine the nuclear and cell size of GSCs in three-dimensional type 1 collagen hydrogels. These measurements were compared to similar measurements taken of established differentiated (bulk) U-251 MG GBM cells and nonmalignant human astrocytes (NHAs) as well as nonmalignant human neural stem cells (NSCs) (Figure 1).

As seen in Figure 1(b), within the 3D collagen hydrogels the nuclear sizes of the GSC populations (GBM10, VTC-061, and VTC-064) and U-251 GBM cells are significantly larger than the healthy NHAs. The GSCs have a nuclear size similar to those of U251 bulk tumor cells. Interestingly nonmalignant NSCs exhibit cell morphologies similar to malignant GSCs with nuclear areas larger than healthy astrocytes. Because of their spherical shape, the GSCs and NSCs both have substantially less spreading and therefore a smaller cytoplasmic area than bulk tumor cells or astrocytes. This morphological feature is reflected in their nuclear-to-cytoplasm ratio, which is significantly greater for GSCs and NSCs than that of either bulk tumor cells or healthy cells (Figure 1(c)).

### 2.2. GSCs Are Variably Sensitive to H-FIRE-Induced Cell Death

**Figure 2:**
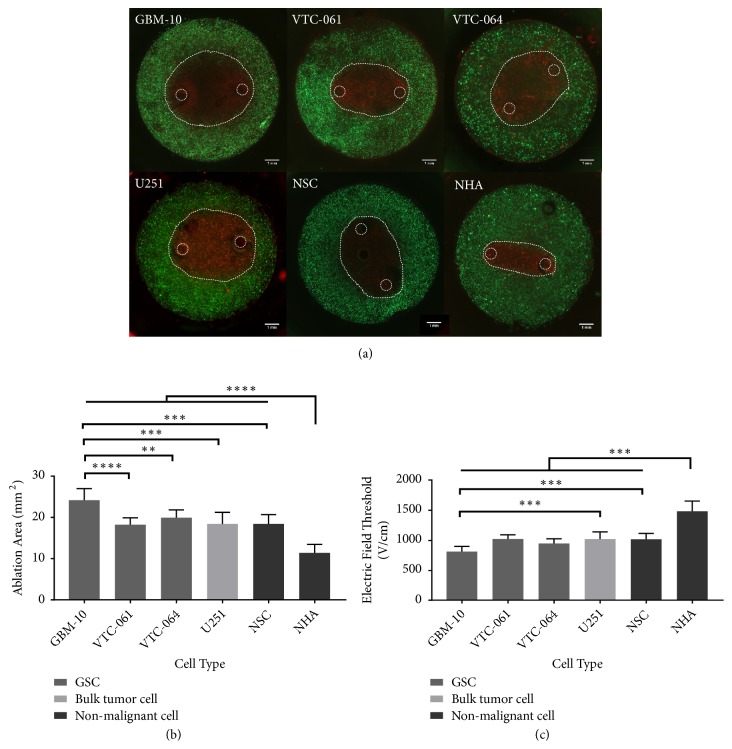
*Populations of patient-derived glioma stem cells are responsive to H-FIRE therapy at variable but lower thresholds than healthy astrocytes.* (a) A visible lesion was created in collagen hydrogels seeded with cells. Electrode placement and ablation lesion outlined by dotted white line (b) Comparison of lesion areas shows three GSC populations have greater lesion sizes than healthy astrocytes and similar lesion sizes to bulk tumor cells and NSCs (c) GSC populations have a lower lethal threshold than healthy astrocytes and similar thresholds to U251 cells and NSCs when exposed to H-FIRE pulses. ^*∗∗*^p < 0.01, ^*∗∗∗*^p < 0.001, and ^*∗∗∗∗*^p < 0.0001.

Three different populations of patient-derived GSCs (GBM 10, VTC-061, and VTC-064) were seeded in 3D collagen hydrogels and exposed to H-FIRE therapy with pulse waveforms of 0.5 *μ*s-2 *μ*s-0.5 *μ*s (Figure 7). For all GSC populations an ablation zone (lesion) was produced by exposing the hydrogel to H-FIRE pulses (Figure 2(a)). To understand the effect of H-FIRE on GSC populations, these ablation areas were compared to the ablation areas for U-251 differentiated tumor cells, NHAs, and NSCs. The ablation areas of all three GSC populations tested were significantly greater than the ablation area of NHA-seeded hydrogels (p < 0.0001) (Figure 2(b)). Only one GSC population, GBM 10, had a significantly larger ablation area than U-251 established GBM cells (p = 0.0002). GBM-10 cells also had a significantly greater ablation area than the NSCs (p = 0.0002). Yet the NSCs had an ablation area similar to the other malignant cell types, significantly greater than the ablation area for NHAs. The GBM-10 cell population had significantly greater ablation area than VTC-061 (p < 0.0001) and VTC-064 (p = 0.0012) as well.

As seen in Figure 2(c), the larger ablation areas of GSCs, U-251s, and NSCs correspond to lower lethal electric field thresholds compared to healthy astrocytes. For the GBM-10 population treated with H-FIRE delivering a 0.5 *μ*s-2 *μ*s-0.5 *μ*s waveform, the lethal threshold is 816 ± 84 V/cm. For VTC-061 cells, the lethal threshold is 1019 ± 70 V/cm. For VTC-064 cells the lethal threshold is 949 ± 73 V/cm. The lethal threshold for the GBM-10 cell population is significantly lower than the lethal threshold for U-251s which have a lethal threshold of 1020 ± 120 V/cm (p = 0.0002). The VTC-061 and VTC-064 GSC populations treated with a 0.5 *μ*s-2 *μ*s-0.5 *μ*s waveform are not significantly different than U-251 bulk cells nor the NSCs. The NSCs had a lethal threshold of 1015 ± 99 V/cm, similar to the bulk tumor cells and the VTC-064 and VTC-061 cell populations. The lethal threshold for NHA is significantly greater than the thresholds for the GSCs, NSCs, and U251s at 1480 ± 175 V/cm (p < 0.0001).

**Figure 3:**
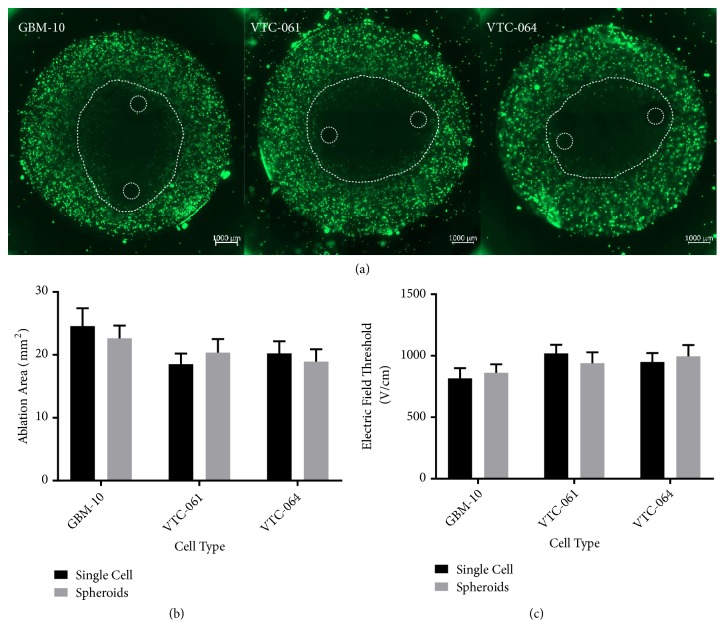
*GSCs grown in multicellular spheroids in hydrogels exhibit similar H-FIRE response to single cells seeded in hydrogels*. (a) H-FIRE lesions for GSCs grown into multicellular spheroids. (b) Comparison of single cell and spheroid hydrogels shows no significant difference in H-FIRE ablation areas. (c) Lethal electric field thresholds for treatment of GSCs do not change when treating spheroids versus single cells in collagen hydrogels.

Because GSCs grow in tumorspheres that can reach hundreds of cells in size, we next determined the lethal threshold of cells in such large tumorspheres to determine if the bunched cell morphology changes response to H-FIRE. GSCs were cultured in hydrogels for 9 days and exposed to H-FIRE pulses. This time-scale allowed cells to grow into large neurospheres within the collagen hydrogels. The spheroid clusters exhibit no significant difference in H-FIRE ablation area from single cell GSC hydrogels (Figure 3). From these results it can be concluded that the growth of the studied cells in 3D-spheroids does not affect GSC response to H-FIRE treatment.

### 2.3. Hyaluronic Acid Scaffolds Induce an Altered Cell Morphology

**Figure 4:**
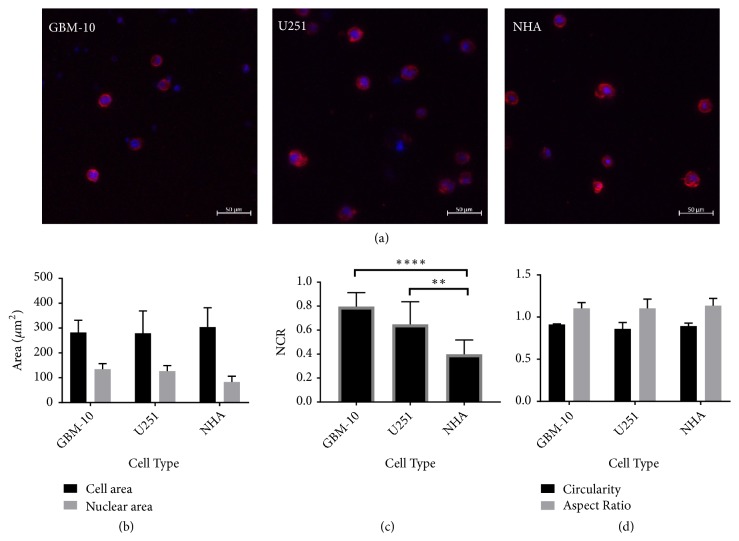
*Cells seeded in hyaluronic acid hydrogels exhibit more uniform morphologies compared to collagen-seeded cells*. (a) Confocal images of cells seeded in HA hydrogels show a rounded and compact cell morphology regardless of cell type. (b) Cells seeded in HA have similar cell areas (p > 0.05) while malignant cells have larger nuclear areas than healthy cells (p < 0.01). (c) GSCs and U251 cells exhibit a larger NCR than NHAs: ^*∗∗*^p < 0.01; ^*∗∗∗*^p < 0.001. (d) All three cell types exhibit similar circularity (p > 0.05) and aspect ratios (p > 0.05).

We next conducted control experiments with the goal of providing a more brain-relevant extracellular matrix (ECM) environment. To accomplish this, we seeded each representative cell type—GSC, bulk tumor cell, and nonmalignant astrocyte—into hydrogels composed of hyaluronic acid (HA). HA is a major component of brain ECM and is upregulated in GBM tumors [54, 55]. HA is important in maintaining the stem cell phenotype of GSCs and has been shown to better mimic* in vivo* biological and clinical behavior of GSCs in terms of both stem cell expression and drug response [56, 57]. Therefore, we chose HA as the biomaterial for these experiments. Cells seeded within the HA all exhibited a highly rounded morphology (Figure 4(a)). Because GBM-10 cells exhibited the lowest cell death electric field threshold in collagen among the patient-derived cells studied, these were selected for continued experiments in the HA scaffolds. In this context the GSC population, GBM 10, had similar overall cell areas to the bulk tumor cells, U-251, and the nonmalignant NHA cells when seeded within the HA (Figure 4(b)), thereby simplifying comparisons among these cell types. The nuclear areas of the malignant cells were all significantly greater than the nonmalignant NHAs. This results in NCR values for both GBM-10 and U-251 significantly greater than NHAs (Figure 4(c)). Unlike when seeded in collagen, the different cell types seeded in HA hydrogels all exhibit highly rounded morphology with similar circularities and aspect ratios (Figure 4(d)). Because cells seeded in HA all exhibit a morphology similar to the GSCs in collagen, while maintaining the trends of NCR and nuclear size, these hydrogels were used to test the robustness of the trends seen in collagen hydrogels treated with H-FIRE.

### 2.4. GSC Selectivity Trends Hold in HA Scaffolds

**Figure 5:**
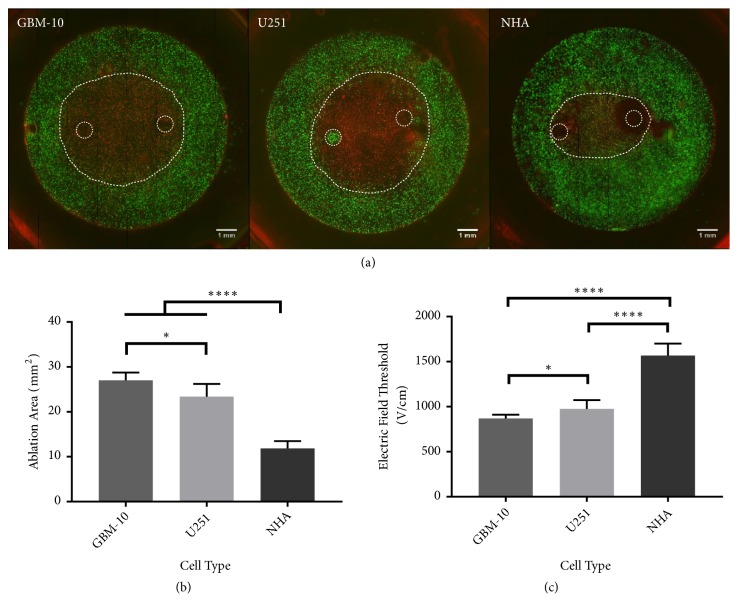
*The ablation of GBM-10 GSCs is accomplished at lower thresholds than other cell types in hyaluronic acid.* (a) Lesion areas after H-FIRE electroporation protocols in HA are visualized with live/dead staining. (b) Comparison of lesion areas in HA shows the GBM-10 GSC populations have a greater lesion size than U251s which has a greater lesion size than NHAs. (c) GSC populations in HA have a lower lethal threshold than healthy astrocytes and slightly lower but similar thresholds to U251 cells when exposed to H-FIRE pulses. ^*∗*^p < 0.05; ^*∗∗∗∗*^p < 0.0001.

To test the effect of the altered ECM and resulting cell phenotype and morphology on electric field thresholds with H-FIRE therapy, cell-seeded HA scaffolds were exposed to electroporation protocols (Figure 5(a)). The ablation area achieved when GBM-10 scaffolds were exposed to H-FIRE was significantly greater than the ablation area of U-251 cells. Both GBM-10 cells and U-251 cells exhibited a significantly larger ablation area than NHA cells. The electric field thresholds derived from the ablation areas are 869 ± 42 V/cm for GBM-10 cells, 976 ± 97 V/cm for U-251 cells, and 1568 ± 133 V/cm for NHA cells. The selectivity trends seen in collagen remain for cells seeded in HA as GBM-10 lethal electric field thresholds are significantly lower than both U251 and NHA cells, while U-251 cells have a significantly lower lethal threshold than NHAs.

**Figure 6:**
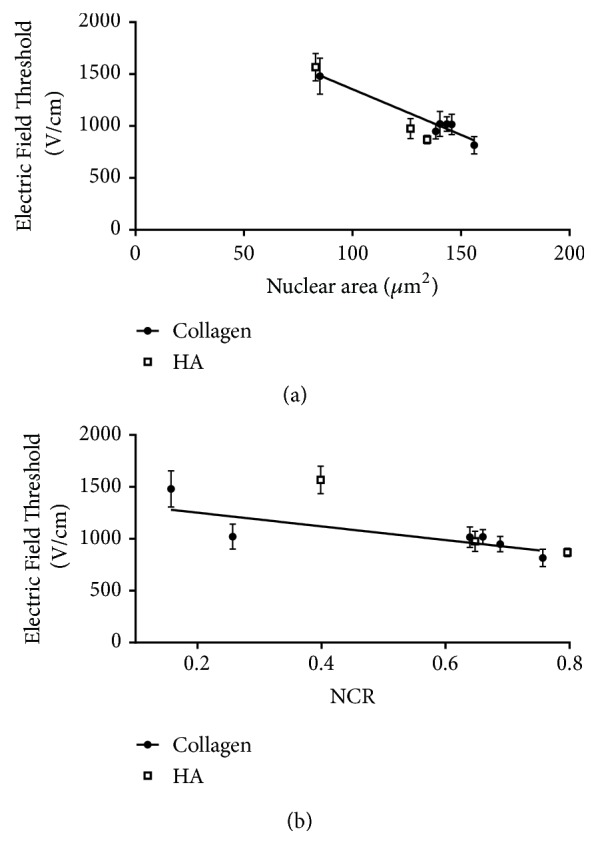
*Summary of data in collagen and HA scaffolds.* (a) A clear relationship emerges between nuclear area and lethal electric field threshold in both collagen and HA where cells with larger nuclei are ablated at lower electric field thresholds (r^2^=0.89 linear correlation). (b) The relationship between NCR and electric field threshold is described by a reciprocal relationship, but with a significantly weaker dependence on NCR (r^2^=0.57 linear correlation).

**Figure 7:**
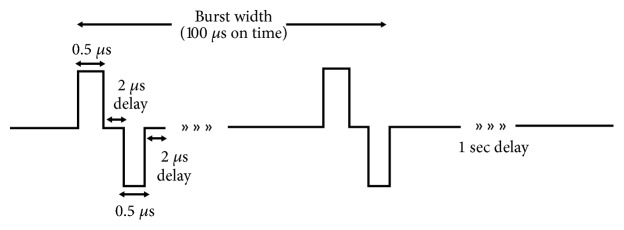
*Schematic of H-FIRE waveforms used in the study.* Collagen scaffolds were treated with a series of 50 bursts delivered at a frequency of one burst/second and amplitude of 800 V for a total on time of 100 *μ*s. HA scaffolds were treated with the same parameters at an applied voltage of 950 V.

We next combined results from both the collagen and the HA scaffolds into a single graphical summary (Figure 6). Plotting lethal electric field threshold versus nuclear area shows a close correlation between the two variables with cells with larger nuclei requiring less energy to ablate, across all cell types (Figure 6(a)). This trend holds even when the scaffold material is changed and overall cell morphology is changed significantly as a result. The relationship between lethal electric field threshold and NCR has an overall similar trend with a decreasing electric field necessary for ablation as NCR increases. However, this dependence is not as strong as is observed for the dependence on nuclear area, and there were some cases where significant variation in NCR did not result in altered electric field threshold (Figure 6(b)).

## 3. Discussion

The results of our work determine that the GSC cells lines studied here are susceptible to H-FIRE ablation to a similar degree as differentiated tumor cells and are significantly more susceptible than normal astrocytes. Interestingly, two of the GSC populations tested (VTC-061 and VTC-064) have been shown previously to be resistant to chemotherapeutics [58]. Therefore, the ablation of these cell populations represents a potential means of targeting radiation and chemotherapy resistant cells that may repopulate tumors if found* in vivo*. However, based on our results, NSCs present in the immediate surroundings of the tumor space may be undesirably targeted by H-FIRE treatment. NSCs reside mostly in the hippocampus and the subventricular zone of the adult brain [59–61]. Hence, H-FIRE pulses must be applied with care in these areas. Our findings highlight the importance of considering the impact on tissue stem cell populations when utilizing H-FIRE procedures in other tissues.

In our previous studies [58, 62, 63], we isolated and characterized glioblastoma stem cells from patient specimens. GSCs were cultured using sphere-forming assay. Next, the stem cell identity of these primary lines was verified using cell dilution assay and differentiation assay. By probing GBM stem-like cell lines with nestin (a well-established stem cell marker for GBM stem-like cells), we found that nestin was differentially expressed among glioblastoma stem-like cell lines and the levels of nestin did not correlate with the capability of GBM stem-like cells to self-renew. Our results strongly suggest that glioblastoma stem cells are heterogenous. Using sphere-forming assay for isolating GSCs does not guarantee the purity of GSC culture. However, given the lack of definitive markers for cancer stem cells from GBM, we argue that sphere-forming assay would be more reliable than marker-based cell sorting. For example, several prior reports show that CD133 negative glioblastoma cells (a classical cancer stem cell marker) also exhibit stem-like cell characteristics [64–66]. As such, sphere-forming assay retains heterogeneity within the populations of GBM stem-like cells, which may provide a better comparison between heterogenous GBM stem-like cells and astrocytes. In order to acquire unbiased results, we used heterogenous GBM stem-like cells in this study to compare the effect of H-FIRE between tumor and normal cells. Whether the properties of GSCs or their genetic heterogeneity results in the differences in H-FIRE treatment we observed that further investigation will be required.

We have previously suggested that both nuclear size as well as NCR may be important regulators of cell death in response to H-FIRE, as both of these parameters were previously shown to correlate with measured electric field cell death thresholds [48, 67]. Interestingly, our comparison between collagen and HA scaffolds provided some test cases in which there was a large change in NCR yet with minimal change in lethal threshold observed. For example, U-251 cells cultured in collagen have an elongated morphology and therefore have a smaller NCR than when cultured in HA, in which the U-251s have a compact morphology. Yet U-251 cells have similar H-FIRE thresholds in both of these contexts, and in both instances exhibit a lower electric field threshold for ablation than NHAs. This result is seemingly difficult to reconcile with our previous results demonstrating enhanced H-FIRE response with an ephrin signaling-induced increase in NCR for malignant cells [67]. While our current results confirm a general trend between electric field threshold and NCR, these results suggest a stronger relationship between electric field threshold and nuclear area. In the case of comparing thresholds in collagen and HA, cell-ECM interactions may furthermore play an important role, making comparison across scaffolding more challenging. Additionally, other signaling changes upon ephrin-activation may also be important in the context of IRE or H-FIRE induced cell death (e.g., altered cytoskeleton integrity [68]), such that the ephrin effect on thresholds observed in previous work may not have been purely due to morphological changes. It should also be noted that, while cell size and morphology are crucial factors in determining the onset of electroporation, they are not the only important aspects for resulting cell death thresholds. Cells are permeabilized based on their morphology and 3D orientation; however, cell viability is also a function of their susceptibility to loss of homeostasis. These complex relationships between morphology and electric field threshold [69, 70] suggest that future work needs to include additional normal cell types with large nuclei as well as tumor cells with small nuclei, with the understanding that a range of cellular or tissue characteristics (e.g., metabolism, extracellular ionic concentrations [71, 72], and tissue conductivity) may be important additional factors in regulating cell death.

It is important to note that while the GSCs presented here exhibit the enlarged NCR seen* in vivo* [52], the morphology of these cells in the collagen hydrogel does not reflect the morphology of these cells* in vivo*. This is not especially surprising, as tumor stem cells tend to propagate as spheroids when cultured* in vitro*. Importantly for this study, the hydrogel platform was able to provide a 3D environment where cells experienced a range of electric fields, much like they would during an* in vivo* treatment. In addition, lethal thresholds can be easily determined using finite element modeling. However, collagen is not an ideal material for recapitulating stem cell properties found* in vivo*. Therefore, cells were also tested in hyaluronic acid hydrogels, a more physiologically relevant material [56, 57]. Recreating the morphology of GSCs using* in vitro* models remains a challenge. Cells in our HA cultures retained spherical morphologies that are not representative of* in vivo* tissues but that are similar to other spheroid stem cell culture methods. Despite this lack of physiological relevance for GSC morphology in our hydrogel tissue mimics, the high NCR exhibited in our* in vitro* cultures of GSCs has been confirmed* in vivo* [52, 53, 73]. HA experiments furthermore provided a method to confirm that the lower electric fields necessary for ablation of GSCs in collagen gels were not due to a rounded morphology that may not be found* in vivo*. In HA scaffolds, GSCs, bulk tumor cells, and healthy astrocytes all take on a similar rounded morphology; however cell type-specific variations in cell death thresholds remained as in collagen. Therefore, we can conclude that the low electric field thresholds seen with GSC cells are not due to their circular morphology and may well be conserved if they were to take on a different morphology* in vivo*. However future* in vivo* experiments will be necessary to test this hypothesis.

The results of this study confirm and expand on our previous results demonstrating that a lower H-FIRE threshold is required for ablation of malignant cells compared with important bulk cell types in the context of GBM. The results of this study add robustness to this conclusion as all previous malignant cells used were established and differentiated cell lines. This study demonstrates the feasibility of using patient-derived cells for therapy testing to determine the different thresholds needed for various patient samples. The results of this study may be of high clinical relevance because they suggest an option for treatment of cells currently considered therapy-resistant. We have demonstrated successful and selective ablation of two populations of cells previously shown to be highly resistant to chemotherapy (VTC-064 and VTC-061) [23, 58]. These results suggest that H-FIRE may be a valuable therapy to be used in conjunction with more traditional therapies to reduce the population of resistant cells that may be left behind to cause tumor recurrence. Studies have shown cancer stem-like cells usually exist in a quiescent state but the population may grow exponentially when stimulated by surgery, chemotherapy, and radiotherapy [74]. Further studies will be done to ensure H-FIRE does not produce an increase in growth kinetics of these GSCs. If no such effect is found, it may be a valuable practice to follow surgery, chemotherapy, and radiotherapy used in GBM treatment with a regime of H-FIRE pulses. This may protect against GSCs causing a recurrent tumor as a result of therapy resistance and increased growth.

## 4. Materials and Methods

### 4.1. Cell Culture

GBM-10, VTC-061, and VTC-064 patient-derived glioma stem cells were received from the lab of Dr. Zhi Sheng. These cells were isolated from resected tumor tissue as described previously [62]. GBM-10, VTC-061, and VTC-064 cells were cultured as free-floating neurospheres in Dulbecco's Modified Eagle Medium (DMEM) (ATCC, Manassas, VA) supplemented with B27 (Life Technologies, Carlsbad, CA), 20 ng/mL epidermal growth factor (EGF) (Life Technologies, Carlsbad, CA), 20 ng/mL basic fibroblast growth factor (bFGF) (Life Technologies, Carlsbad, CA), 1% L-glutamine (Life Technologies, Carlsbad, CA), and 1% Penicillin/Streptomycin (PS) (Lonza, Basel, Switzerland). Normal Human Astrocyte (NHA) cells (Lonza, Basel, Switzerland) were cultured in Astrocyte Growth Media (Lonza, Basel, Switzerland). U-251 MG human glioblastoma cells (ATCC, Manassas, VA) were grown in DMEM containing 10% FBS, 1% PS, and 0.1 mM nonessential amino acid. Human Neural Stem cells (NSCs) were cultured as nonadherent neurospheres in StemPro™ NSC SFM (Thermo Fischer Scientific, Waltham, MA). All cells were grown in culture at 37°C in 5% CO_2_ in a humidified incubator. Cells were seeded in hydrogels at a density of 1 × 10^6^ cells/mL. The hydrogels were submerged in appropriate growth media for the cell type at 37°C in 5% CO_2_ in a humidified incubator and cell viability was maintained within hydrogels for up to 14 days.

### 4.2. Construction of Collagen Scaffolds

Stocks of type I collagen were prepared by dissolving rat tail tendon in acetic acid, followed by freezing and lyophilization as described previously [75]. Stock solution concentrations of collagen were created at a density of 10 mg/mL. Scaffolds with a final concentration of 5 mg/mL were made from concentrated collagen stocks to create collagen gels of 0.5% (w/w). Neutralized collagen solutions were created by mixing acid-dissolved collagen with 10X DMEM (10% of total collagen solution volume) and sufficient volumes of 1N NaOH until a pH in the range of 7.0–7.4 was achieved. The neutralized collagen was mixed with cells suspended in DMEM, NHA, NSC, or GSC media to achieve a cell density of 1 × 10^6^ cells/mL in the final collagen mixture. Solutions were mixed carefully with a sterilized spatula to ensure homogenous distribution throughout the gel without damaging cells. Collagen solutions were then dispensed into a polydimethylsiloxane (PDMS) mold with a cut-out of 10 mm diameter and 1 mm depth and molded flat to ensure consistent scaffold geometry. Our previous mathematical modeling and experiments on oxygen (O_2_) consumption rates by tumor cells [76] confirm that, at this cell density and scaffold thickness, O_2_ concentration is uniform throughout the scaffold depth. Collagen was allowed to polymerize at 37°C and 5% CO_2_ for 30 minutes. For testing of GSCs neurosphere morphology, hydrogels were seeded with single cells at a cell density of 1 × 10^5^ cells/mL and maintained in culture for 9 days to allow time for large neurospheres to grow from the individual cells.

### 4.3. Construction of Hyaluronic Acid Scaffolds

GBM-10, U251, and NHA cells were cultured in hyaluronic acid Hystem-C hydrogels (ESI Bio, Alameda, CA) according to the manufacturer's instructions. Prior to fabricating the 3D HA scaffolds, cells were removed from their flask using trypsin (Life Technologies, Carlsbad, CA) and centrifuged at 900 rpm for 6 min to form a cell pellet. Cells pellets and resuspended in an equal parts mixture of glycosil (thiol-modified sodium hyaluronate) and Gelin-S (thio-modified gelatin). To form a hydrogel, the solution was mixed with Extralink (PEGDA, polyethylene glycol diacrylate) and allowed to crosslink in a 37°C incubator with 5% carbon dioxide for 30 minutes. After hydrogels were crosslinked, appropriate culture media for the seeded cell type were added to the well and gels were placed in the incubator and maintained until treatment. Cells were seeded in the Hystem-C hydrogel to create a final concentration of 1x10^6^ cells/mL.

### 4.4. Fluorescent Staining

GBM-10, VTC-064, VTC-061, U251, NSC, and NHA cells were separately seeded in hydrogels described earlier. After culturing the cells for 24 hours, the hydrogels were fixed using 4% formalin and permeabilized and blocked using 40 mg/mL bovine serum albumin (BSA) and 0.05% Triton-X. Cellular F-actin was stained with Alexa Flour 568 phalloidin (Life Technologies, Carlsbad, CA) while cell nuclei were stained with diaminophenylindole (DAPI; Sigma-Aldrich, St. Louis, MO). Cells were visualized using a Zeiss LSM880 (Carl Zeiss Microscopy LLC, Thornwood, NY) laser scanning confocal microscope.

### 4.5. Determination of NCR

Fluorescent stained cells were used to determine overall cell area and nuclear area for cells. Image analysis was done in Image J (NIH, Bethesda, MD). Z-stack images were converted into 2D projection images and cell measurements were made from these projections. Measurements were made on at least four cells from different area of the hydrogel (20 cells total) and at least 5 hydrogels were analyzed for each condition. NCR was calculated from the measured cell area (A_C_) and nuclear area (A_N_) as follows:

(1) $$\begin{array}{c} NCR=\frac{A_{N}}{A_{C}-A_{N}} \end{array}$$

Aspect ratio was calculated from the measured largest diameter (dmax) and smallest diameter orthogonal to dmax (dmin) as follows:

(2) $$\begin{array}{c} A_{R}=\frac{d_{min}}{d_{max}} \end{array}$$

Circularity of the cells was calculated from the measured perimeter of the cell (P) and overall cell area (A_C_) as follows:

(3) $$\begin{array}{c} f_{circ}=\frac{4\pi A_{C}}{P^{2}} \end{array}$$

The scaffolds that all cells were seeded in are isotropic and therefore there was no observed orientation dependence on this 2D projection from the 3D morphology.

### 4.6. Electroporation of 3D Scaffolds

Pulsed electroporation experiments were performed in hydrogels with constant electrical properties. The electrical conductivities of each of the collagen gel-cell mixtures were measured before crosslinking, using a conductivity meter (B-173, Horiba, Kyoto, Japan) to ensure similar electrical properties (0.98 ± 0.04 S/m). H-FIRE pulses were delivered using a custom-built pulse generation system (INSPIRE 2.0, VoltMed Inc., Blacksburg, VA). Media were aspirated from hydrogels prior to exposure to treatment. Two solid stainless-steel cylinders with diameters of 0.87 mm, separated 3.3 mm edge-to-edge, were used as electrodes. These electrodes were inserted through the full thickness of the hydrogel. These electrodes were connected to the pulse generation system, which was programmed to deliver treatment according to the H-FIRE protocol. For the H-FIRE protocol used, bursts consisting of 200 × 500 ns pulses with a 2 *μ*s interpulse delay were delivered with a repetition rate of 1 burst per second for a total of 50 bursts. For cells seeded in collagen, a peak voltage of 800 V was used to produce ablations in the hydrogel large enough for distinct electric field lines to be measured but small enough that boundary effects of the hydrogel edge did not interfere with the electric field distribution at the lethal threshold. In treating cells seeded in HA hydrogels, a peak voltage of 950 V was used.

### 4.7. Finite Element Analysis in Hydrogels

**Figure 8:**
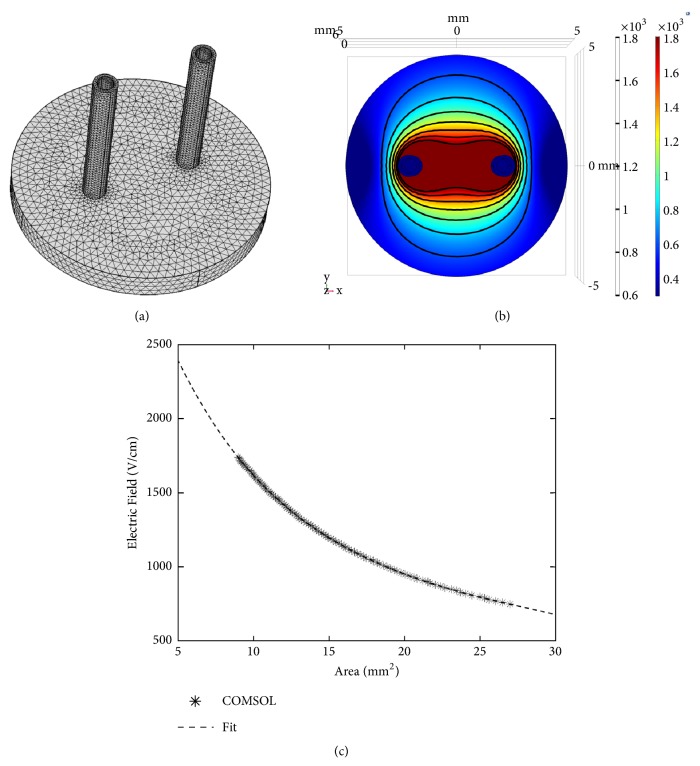
*Finite element model relates ablation area to electric field threshold in hydrogel system.* (a) Geometry and meshing of the finite element model used to simulate treatment of the hydrogel platform. (b) COMSOL model of hydrogel exposed to H-FIRE treatment (800V) shows how electric field changes across the scaffold. (c) Numerical integration of the finite element model of the electric field distribution in the scaffold allows for correlating ablation area to lethal electric field threshold.

Finite element models using COMSOL Multiphysics (Version 4.3, COMSOL Inc., Palo Alto, CA) were used to solve the Laplace equation to find the electric field distribution within the hydrogels for each different voltage used. COMSOL Multiphysics was also used to solve the Joule heating equation to calculate the temperature distribution in the hydrogel as a result of each treatment. The simulation geometry was modeled as a 10 mm diameter and 1 mm thick cylinder with two steel electrode cylinders (d = 0.87 mm) spanning the depth of the hydrogel (Figure 8(a)). Thermal and electrical properties for each domain have been described previously [77]. The mesh was refined until error between successive refinements was less than 1%. The final mesh contained 47,438 elements and solutions were found in approximately 3 minutes on a Pentium i3 processor.

### 4.8. Determination of Lethal Threshold

The thresholds for cell death were determined by analyzing images taken of live-dead stain on the hydrogels 24 hours after delivering treatment. Hydrogel scaffolds were incubated in 2 *μ*M Calcein AM (Biotium, Hayward, CA) and 4 *μ*M ethidium homodimer III (Biotium, Hayward, CA) in PBS for 30 minutes at room temperature. Live cells were labeled green with calcein AM staining while cells lacking membrane integrity were labeled red with ethidium homodimer III and were considered dead. Images of each hydrogel were captured used a Zeiss LSM 880 microscope using a 5X objective and pieced together by automatic stitching. A custom algorithm developed in MATLAB was used to measure the lesion area from the imported images. Lesion areas were determined by analyzing intensity of points from the green channel in MATLAB as described previously [78]. A threshold of 20% of the maximum intensity value was used to separate the live region from the dead region in each image. Geometric measurements of the ablation zones were mapped to a finite element model developed in COMSOL Multiphysics 5.3 (Stockholm, Sweden) as described previously [48] (Figure 8(b)). Numerically integrating the surface of the scaffold across a range of electric field magnitudes in the finite element model allows for correlating ablation area to lethal electric field threshold (Figure 8(c)). A sixth-order polynomial found with least squares fitting in MATLAB resulted in a maximum relative error of 4.7% between electric field from the numerical fit and the COMSOL model. Use of this polynomial fit allows for a calculation of the electric field threshold for each ablation area from the COMSOL model for the scaffold. The lethal electric field threshold reported is the electric field distribution at the edge of the measured lesion area for the designated electrode and hydrogel configuration. It should be noted that the thresholds calculated here show the minimum field needed to cause cell death. The minimum field needed to induce electroporation is not calculated here.

### 4.9. Statistical Analysis

Statistical significance was determined by a two-tailed* t-*test performed in Prism Statistical Software (Version 6, Graphpad, La Jolla, CA). A 95% confidence interval was used with significance defined as p < 0.05. All numerical results are reported as the mean and the standard deviation of all experimental measurements. No outliers were excluded.
